# Wild Boars as a Reservoir of Zoonotic Hepatitis E Virus in Portugal with Full-Genome Evidence of Genotype 3m

**DOI:** 10.3390/pathogens15040430

**Published:** 2026-04-16

**Authors:** Bernardo Almeida, Inês Caetano, Margarida Santos, Ana Duarte, Margarida Dias Duarte, Sílvia Carla Barros, Fábio A. Abade dos Santos, Ana Margarida Henriques

**Affiliations:** 1 National Institute of Agrarian and Veterinarian Research, Quinta Do Marquês, Av. da República, 2780-157 Oeiras, Portugal; bsa.almeida2002@gmail.com (B.A.); ines.caetano@iniav.pt (I.C.); margarida.marques@iniav.pt (M.S.); ana.duarte@iniav.pt (A.D.); margarida.duarte@iniav.pt (M.D.D.); silvia.santosbarros@iniav.pt (S.C.B.); fabio.abade@iniav.pt (F.A.A.d.S.); 2 Centre for Interdisciplinary Research in Animal Health (CIISA), Faculdade de Medicina Veterinaria, Universidade de Lisboa, Avenida da Universidade Tecnica, 1300-477 Lisboa, Portugal; 3 Associate Laboratory for Animal and Veterinary Sciences (AL4AnimalS), Avenida da Universidade de Lisboa, 1300-477 Lisboa, Portugal; 4 Departamento de Ciências da Vida, Faculdade de Ciências e Tecnologia, Universidade Nova de Lisboa, Campus da Caparica, 2829-516 Caparica, Portugal; 5 CECAV—Centro de Ciência Animal e Veterinaria, Faculdade de Medicina, Veterinaria de Lisboa, Universidade Lusofona, Centro Universitario de Lisboa, 1749-024 Lisboa, Portugal

**Keywords:** Hepatitis E virus (HEV), wild boar, one health, zoonotic transmission, phylogenetic analysis, phylogeographical analysis, recombination analysis

## Abstract

Hepatitis E virus (HEV) is a zoonotic pathogen of global concern that circulates in both domestic and wild swine populations. Understanding its presence and dynamics in wildlife reservoirs is crucial for assessing spillover risks and designing One Health surveillance strategies. This study investigated the occurrence, genetic diversity, and evolutionary relationships of HEV in wild boars from mainland Portugal. A total of 120 animals from seven districts were tested, with HEV RNA detected in four cases (3.3%), all from the Évora district near the Spanish border. One positive sample was successfully sequenced, and phylogenetic analysis based on the complete genome classified it within the HEV-3m subtype, clustering with predominantly human-derived sequences from Spain and France, which highlights its zoonotic potential. A second phylogenetic analysis based on a partial genomic fragment, including sequences from domestic pigs in Portugal, revealed the co-circulation of subtypes 3e, 3f, and 3m without clear spatial or temporal patterns. Phylogeographic analysis suggested that the identified strain was most likely introduced from Spain, supporting the hypothesis of cross-border transmission through wild boar movement. No recombination events were detected in the sequence obtained in this study. These findings provide the first molecular evidence of HEV-3m circulation in wild boars in Portugal, offering valuable insight into the HEV strain circulation in European wildlife populations. The zoonotic potential of HEV and the likelihood of interspecies transmission highlight the need for coordinated cross-border surveillance and integrated One Health strategies.

## 1. Introduction

Wild boars (*Sus scrofa scrofa*) have expanded their populations across Europe in recent decades [1]. They act as important reservoirs of pathogens and pose an increasing risk of spillover to other species, such as domestic pigs and humans, due to the zoonotic potential of some of these pathogens.

Hepatitis E virus (HEV) is a positive-sense single-stranded RNA virus belonging to the *Hepeviridae* family. To date, eight genotypes (HEV-1 to HEV-8) have been described. HEV-1 and HEV-2 exclusively infect humans [2]. HEV-3 and HEV-4 infect various species, including humans and swine [3]. HEV-5 and HEV-6 have so far only been detected in wild boars in Japan [3,4], while HEV-7 and HEV-8 have been identified in dromedaries in the United Arab Emirates and camels in China, respectively [5,6].

In Europe, HEV-3 is maintained in pig and wild boar populations [7,8,9,10] and can be transmitted to humans through the consumption of undercooked pork or through direct contact with infected animals [11]. HEV-3 genotype is subsequently divided into 14 subtypes, named HEV-3a to HEV-3m and HEV-3ra. Although human infections with HEV-3 are often asymptomatic, they can still cause acute hepatitis and, in immunocompromised individuals, may progress to chronic infection [12,13]. Symptoms may include acute liver failure, which can lead to cirrhosis [14,15,16]. Asymptomatic infection in swine allows the virus to circulate unnoticed, showcasing the importance of surveillance to track its epidemiology.

The prevalence of HEV in wild boar populations across Europe varies substantially between countries and regions. Within the Iberian Peninsula, for example, higher HEV prevalence has been consistently reported in wild boars near the south-west Spanish-Portuguese border [17,18], indicating the presence of localized hotspots where the risk of spillover to humans is consequently higher.

Currently, there are no HEV vaccines available or in use in Europe for humans or animals, and the virus control strategies primarily rely on surveillance programs, good farming hygiene practices and food safety measures, especially thorough cooking of pork products. Given the zoonotic potential of HEV, continuous monitoring of wild swine populations is essential to reduce the risk of transmission to humans and to support effective public health interventions.

To date, no studies of HEV in Portugal have combined epidemiological data with complete genome sequencing alongside phylogenetic, phylogeographic and recombination analyses. Such an integrated approach is crucial for accurately characterizing viral circulation, evolutionary dynamics and potential cross-species transmission risks in wild boars. Therefore, the aim of this study was to address these knowledge gaps by determining the prevalence of HEV in wild boars in multiple Portuguese regions and identifying the circulating genotypes. This also allowed the Portuguese strain to be contextualized within the European and global contexts, and possible routes of introduction and dissemination to be inferred.

## 2. Materials and Methods

### 2.1. Sample Collection

Between October 2023 and February 2025, organ samples were collected from 120 wild boars hunted in seven mainland Portuguese districts, namely Santarém, Évora, Beja, Aveiro, Guarda, Castelo Branco and Portalegre. The tissues collected from the animals, comprised liver, spleen, bone marrow, lungs, retropharyngeal lymph nodes, submandibular lymph nodes, and diaphragm; however, not all of these tissues were collected from every animal. Information on age, health status, and the specific tissues collected from each individual was not recorded at the time of sampling.

### 2.2. Nucleic Acids Extraction

Following handling in a BSL3 laboratory, the samples were homogenized to a concentration of 20% (*w*/*v*) in phosphate-buffered saline (PBS) using a Precellys tissue homogenizer (Bertin Technologies, Montigny-le-Bretonneux, France) and tubes containing zirconium spheres (three homogenization cycles of 20 s each, with 30-s pauses in between), before being clarified by centrifugation at 2000× *g* for 5 min. Nucleic acid extraction was performed using the KingFisher Flex nucleic acid extraction system (ThermoFisher Scientific, Waltham, MA, USA) and the IndiMag Pathogen Kit (Indical, Leipzig, Germany), according to the manufacturer’s protocol.

### 2.3. RT-qPCR for HEV Detection

Reverse transcription quantitative PCR (RT-qPCR), targeting HEV ORF2 and ORF3 overlapping region, was used for HEV detection [19]. The reaction mixture contained 10 µL of RNA template, 1 µM of each primer (50 pmol/µL), 0.2 µM of probe (10 pmol/µL), 1 µL of enzyme and 12.5 µL of buffer (AgPath-ID One-Step RT-PCR Kit from Thermo Fisher Scientific, Waltham, MA, USA). The primer and probe sequences are presented in Table 1.

The RT-qPCR amplification program consisted of a reverse transcription step at 45 °C for 15 min, an initial denaturation at 95 °C for 10 min, followed by 45 cycles of denaturation at 95 °C for 30 s, annealing at 52 °C for 30 s and extension at 72 °C for 30 s. A positive field sample was included in each reaction as a positive control. Non-template negative controls were also included.

### 2.4. Whole-Genome Amplification by RT-PCR

**Figure 1:**
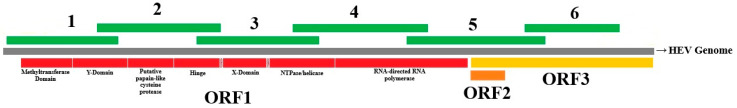
Graphic representation of HEV genome coverage by RT-PCR amplicons used for complete genome sequencing. The gray line represents the HEV genome. Below it, coding regions are shown: the red line corresponds to ORF1, subdivided into its functional domains; the orange line represents ORF2; and the yellow line indicates ORF3. Above the genome, the six green lines represent the overlapping amplicons obtained using specific primer pairs.

One HEV-positive sample from the Reguengos de Monsaraz municipality, in the Évora district, was selected for whole-genome sequencing. Genome amplification was carried out using six different primer pairs, designed to generate overlapping amplicons with a minimum overlap of 120 nucleotides, as shown in Figure 1.

Primers were designed based on a BLAST-derived multiple sequence alignment, initially comprising 1000 HEV genomes retrieved from the NCBI database. Sequences that were incomplete, duplicated or derived from rabbit HEV were excluded, as these lack the ORF1 Hinge and X domains. In addition, a group of sequences originating from China and Japan, characterized by the absence of the Hinge domain and a large portion of the putative papain-like cysteine protease domain, was removed to prevent bias in primer design. Primers were then designed targeting the most conserved regions among the remaining 316 sequences. At each primer position, the nucleotide with the highest frequency was selected. When no nucleotide reached a frequency of at least 80%, degenerate bases were incorporated using the appropriate IUPAC ambiguity codes to represent the predominant variants. Primer sequences are presented in Table 1.

All reactions were carried out using an identical amplification workflow, beginning with cDNA synthesis, using the SuperScript™ III First-Strand Synthesis System (Thermo Fisher Scientific, Waltham, MA, USA). The reaction mixture was prepared with 1 μL of the selected reverse primer (10 pmol/μL), 1 μL of 10 mM dNTP mix and 11 μL of RNA template. This mixture was heated at 65 °C for 5 min and immediately cooled on ice for an additional 5 min. Subsequently, 4 μL of 5× SuperScript III First-Strand Buffer, 1 μL of 100 mM DTT, 1 μL of RNase inhibitor and 1 μL of SuperScript III Reverse Transcriptase were added, for a final volume of 20 μL. cDNA synthesis was then performed under the following conditions: 23 °C for 2 min, 55 °C for 30–60 min and 70 °C for 15 min. After cooling on ice, 1 μL of RNase H was added and the reaction incubated at 37 °C for 20 min.

PCR amplification was conducted using 5 µL of cDNA, 0.5 µM of each primer (50 pmol/µL), 6.5 µM of water and 12.5 µL of NZYTaq II 2x Green Master Mix (NZYTech, Lisbon, Portugal). The qPCR amplification program consisted of an initial denaturation at 94 °C for 3 min, followed by 35 cycles of 94 °C for 45 s, 55 °C for 30 s, 72 °C for 90–150 s and a final extension at 72 °C for 10 min.

Amplification products were observed on 1% agarose gel stained with GreenSafe (NZYTech, Lisbon, Portugal) using a GelDoc Go Imaging System (Bio-Rad, Hercules, CA, USA). Target bands were excised and purified with the NZYGelpure Kit (NZYTech, Lisbon, Portugal).

### 2.5. Sanger Sequencing

Sanger sequencing was performed using BigDye™ Terminator Cycle Sequencing kit (Applied Biosystems, Foster City, CA, USA). Sequencing reactions were set up with the same forward and reverse primers used for PCR amplification. Each 10 µL reaction contained 1 µL of sequencing buffer, 2 µL of sequencing mix, primer at a final concentration of 2.5 µM and a volume of purified PCR product adjusted according to fragment concentration with nuclease-free water added to reach the final volume. Sequencing protocol was carried out with an initial denaturation step at 96 °C for 1 min, followed by 25 cycles of 96 °C for 10 s and 60 °C for 70 s. Sequencing products were purified using the Zymo Research ZR DNA Sequencing Clean-up Kit™ (Zymo Research, Irvine, CA, USA) following manufacturer’s protocol. Purified fragments were sequenced using a 3130 Genetic Analyzer (Applied Biosystems, Foster City, CA, USA) and sequence chromatograms were assembled using SeqScape v2.5 software.

The nucleotide sequence obtained was submitted to GenBank using BankIT and received accession number PX852416.

### 2.6. Phylogenetic and Phylogeographic Analysis

For phylogenetic analysis, sequences were selected ensuring that all HEV genotypes and all HEV-3 sub-genotypes were included. Genotype HEV-3d was not included due to the absence of complete genome sequences available in public databases. Additionally, two rat HEV sequences were included as outliers.

Alignment was performed in AliView v1.28 [20] using Muscle algorithm. Additionally, due to the absence of certain genomic regions from specific groups of sequences, the dataset was further processed using the Gblocks v0.91.1 tool available at https://ngphylogeny.fr/tools/tool/276/form (accessed on 7 January 2026) employing default parameters [21,22]. This processing allowed us to proceed with the phylogenetic analysis without the need to remove from the sequence dataset certain genotypes that lack specific genomic regions, such as genotype HEV-3ra sequences that lack the Hinge Domain and the X-Domain in the ORF1 sequence, or genotype HEV-8 sequences that lack the Hinge domain in its ORF1 sequence. TREE-PUZZLE v5.3 [23,24] was used to assess the phylogenetic signal, ensuring that the dataset was appropriate for phylogenetic analysis. IQ-TREE v3.0.1 [25,26,27] was used, via the command line, to determine the most appropriate nucleotide substitution model for the sequence dataset with the ModelFinder Plus option (-m MFP), which identifies the optimal model according to the Bayesian Information Criterion (BIC). The phylogenetic tree was subsequently inferred under a maximum likelihood framework using the selected model. Nodal support was evaluated using a combination of ultrafast bootstrap analysis and SH-like approximate likelihood ratio tests, each performed with 10,000 replicates. The parameters (-bb 10,000 -bnni -alrt 10,000) correspond to the IQ-TREE command-line options used for this purpose, specifically indicating 10,000 ultrafast bootstrap replicates (-bb 10,000), 10,000 SH-like approximate likelihood ratio test replicates (-alrt 10,000), and the application of bootstrap tree optimization (-bnni). This provides statistical support for the resulting tree topology. A second phylogenetic tree was constructed to include 14 additional 306-nt-long sequences from RT-qPCR-positive HEV samples detected in domestic swine in Portugal in recent years. These sequences were obtained using the system described by Pas et al. (2012) [28], which targets ORF1, and were then Sanger sequenced. Phylogenetic analysis was performed in IQ-TREE using the maximum likelihood method and the TIM2 + F + R3 model (transition model 2). Model selection was performed as indicated above. The resulting trees were visualized using FigTree v1.4.4, with only bootstrap values higher than 70% shown.

Temporal signal was assessed by root-to-tip regression analysis using TempEst v1.5.3 [29] using the phylogenetic tree obtained with IQ-TREE. Phylogeographic reconstruction was performed with the INSaFLU platform v2.2.2 [30,31], which implemented the Nextstrain pipeline, using the same sequence dataset employed in the phylogenetic analysis. Resulting phylogeographic patterns were visualized using Auspice v2.67.0 [32].

### 2.7. Recombination Analysis

Recombination screening was carried out using the RDP4 v4.101 software [33], applying multiple algorithmic approaches to identify potential recombination events, including RDP [34], GENECONV [35], Bootscan [33], MaxChi [36], Chimaera [37], SiScan [38], 3Seq [39], BURT [33], PhylPro [40], VisRD Occupancy [41], TOPAL DSS [42] and LARD [43]. Only recombination signals supported by at least three independent methods and associated with *p*-values below 0.05 were considered reliable. The two Rat HEV sequences were excluded from this analysis.

## 3. Results

### 3.1. HEV Detection

**Figure 2:**
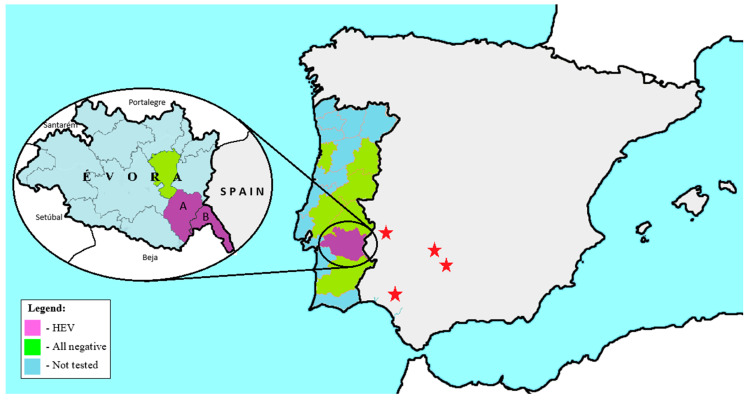
Geographic distribution and prevalence of HEV in wild boars in mainland Portugal. In the left side of the figure, the Évora district was amplified to display the municipalities where HEV-positive wild boars were found. Districts and municipalities are colored based on virus presence: purple for regions with HEV-positive samples (Reguengos de Monsaraz—A, Mourão—B), green for districts and municipalities where only negative samples were found and blue marks untested regions. Red stars indicate the location of HEV hotspots in Spain

Of the 120 wild boars tested, only four were positive in the RT-qPCR screening, three from Reguengos de Monsaraz and one from Mourão. These two municipalities are located in Évora district, near the Portuguese land border with Spain and close to known Spanish HEV hotspots in the wild boar population, as illustrated in Figure 2.

### 3.2. Phylogenetic Analysis

**Figure 3:**
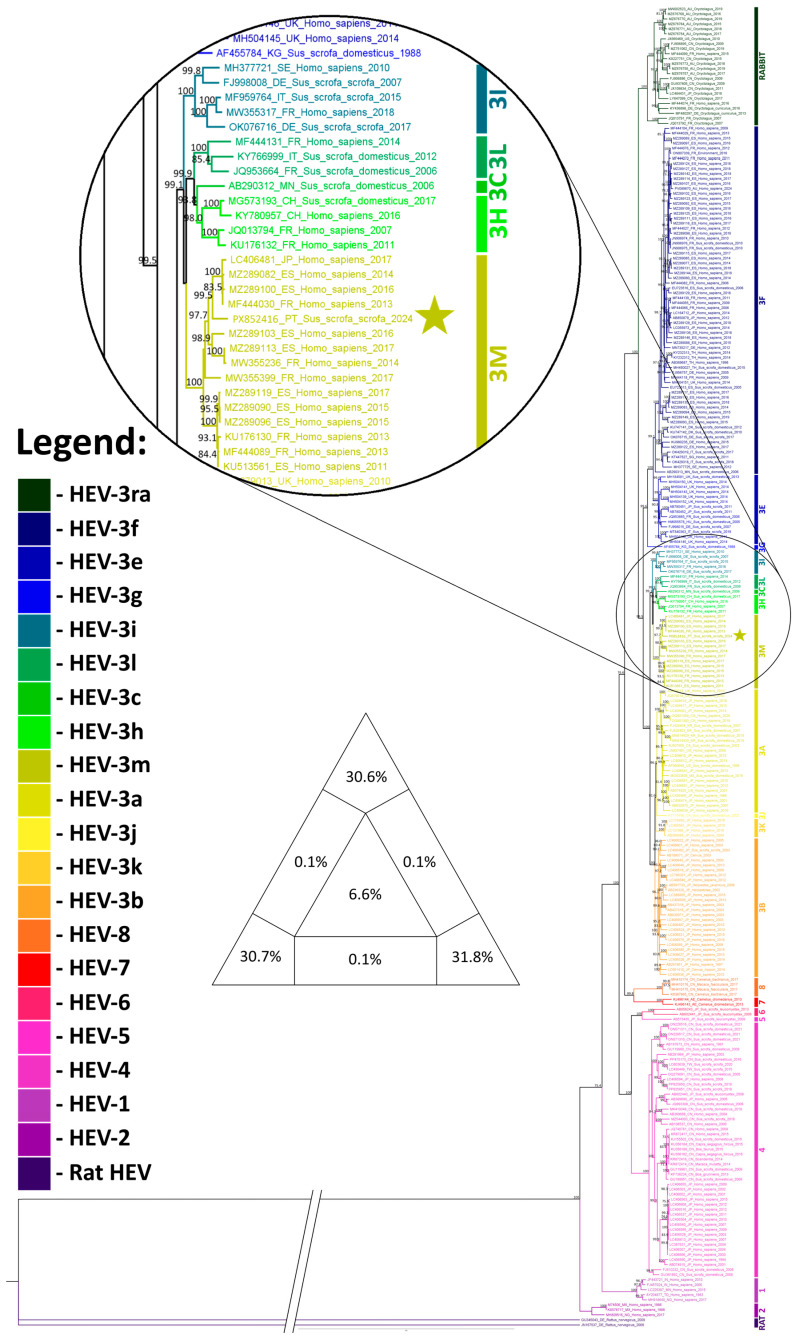
HEV phylogenetic analysis inferred in IQ-TREE by using the Maximum Likelihood method and the best-fit model GTR + F + R6 model (General Time Reversible model). The tree with the highest log likelihood (−221,063.725) is shown. The phylogenetic analysis was performed using the full HEV genome of 265 sequences, with model selection performed by ModelFinder Plus and branch support assessed with 10,000 ultrafast bootstrap replicates and 10,000 SH-aLRT replicates. The sequence marked with a star represents the sample obtained in the present study. Only the bootstrap values higher than 70% are shown in the tree. The triangular diagram on the left side of the phylogenetic tree represents the quartet puzzling support topology obtained with TREE-PUZZLE for this dataset. The percentage of resolved quartets is 93.1% (corresponding to the sum of the three values at the triangle’s tips).

Processing the HEV dataset with the Gblocks reduced the alignment length from 8288 nt to 4980 nt (a 39.9% reduction). Phylogenetic analysis (Figure 3) revealed the expected clustering into the eight main HEV genotypes (HEV-1 to HEV-8). All 13 HEV-3 subtypes recognized to date also formed distinct clades, each supported by strong bootstrap values. HEV genotype 3d is not represented due to the absence of complete genome sequences available from this genotype. The HEV-positive sample sequenced in this study (PX852416) clustered within the HEV-3m clade, which comprises 15 strains in total, 13 of which originating from Spain or France. The other strain originated in Japan. Apart from the sequence obtained in this study from a wild boar, all the sequences in this clade are from humans, identified between 2011 and 2017. This cluster is strongly supported by a bootstrap value of 100. The HEV-3 genotypes were largely divided into two main phylogenetic groups: one group comprising genotypes 3b, 3j, 3a and 3k, consisting predominantly of Asian sequences (87.9%); and another comprising genotypes 3l, 3c, 3h, 3m, 3i, 3g, 3e and 3f, consisting mainly of European sequences (88.5%).

**Figure 4:**
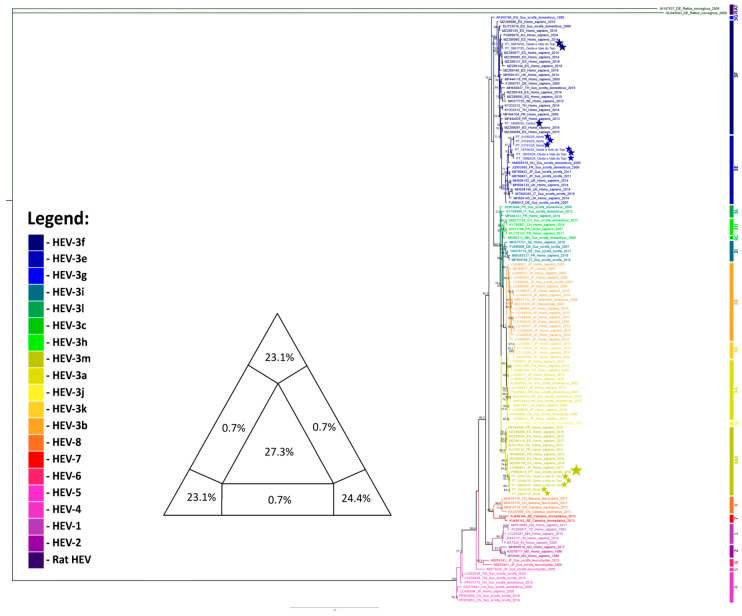
HEV phylogenetic analysis inferred in IQ-TREE by using the Maximum Likelihood method and the best-fit model TIM2 + F + R3 model (Transition Model 2). The tree with the highest log likelihood (−9975.110) is shown. The phylogenetic analysis was performed using partial sequences of HEV genome of 135 sequences, with model selection performed by ModelFinder Plus and branch support assessed with 10,000 ultrafast bootstrap replicates and 10,000 SH-aLRT replicates. The sequence marked with the larger star represents the sample obtained in the present study, while little stars indicate the sequences obtained in domestic swine in Portugal. Only the bootstrap values higher than 70% are shown in the tree. The triangular diagram on the left side of the phylogenetic tree represents the quartet puzzling support topology obtained with TREE-PUZZLE for this dataset. The percentage of resolved quartets is 70.6% (corresponding to the sum of the three values at the triangle’s tips).

A second phylogenetic tree (Figure 4) was constructed using a 306 bp fragment of the ORF1 region, allowing the inclusion of 14 additional sequences from domestic swine on Portuguese farms obtained in a separate study. This approach enabled robust genotyping and comparison of these sequences with reference strains within the established HEV genotype framework. However, the conclusions of this study should be interpreted with caution, given that such short fragments provide limited phylogenetic resolution and are not suitable for drawing robust conclusions about nationwide subtype circulation patterns. Three strains (09816/25, 09817/25, and 18085/25) clustered in genotype 3f, six strains (18744/24, 18918/24, 01191/25, 01192/25, 01193/25, and 10992/25) in genotype 3e, and the remaining five strains (09828/25, 28246/25, 28247/25, 02046/26, and 02047/26), together with the strain characterized in this study, in genotype 3m. The clustering of Portuguese strains from domestic pigs within genotypes 3f, 3e, and 3m is consistent with the previously described division of HEV strains into two main phylogenetic groups (predominantly Asian and predominantly European), with these strains clearly falling into the European group.

There are no apparent temporal or geographical relationships among the strains included within each sub-genotype. For sub-genotype 3f, although only samples detected in mid-2025 are included, these were identified in two regions belonging to different NUTS II areas: Oeste e Vale do Tejo and Centro. In the Oeste e Vale do Tejo region, three additional positive samples belonging to sub-genotype 3e (18744/24, 18918/24 and 10992/25) were detected approximately one year before and at the same time as sub-genotype 3f. Samples belonging to sub-genotype 3e were also detected in the Norte region in early 2025. Regarding sub-genotype 3m, to which the sample from this study belongs (originating from the Alentejo region), samples detected in the Norte and Oeste e Vale do Tejo regions in late 2025 and early 2026 were also included. These findings suggest a dispersed distribution pattern in terms of both time and geographical regions, with no evidence of clustering or localized transmission associated with specific sub-genotypes.

### 3.3. Phylogeographic Analysis

**Figure 5:**
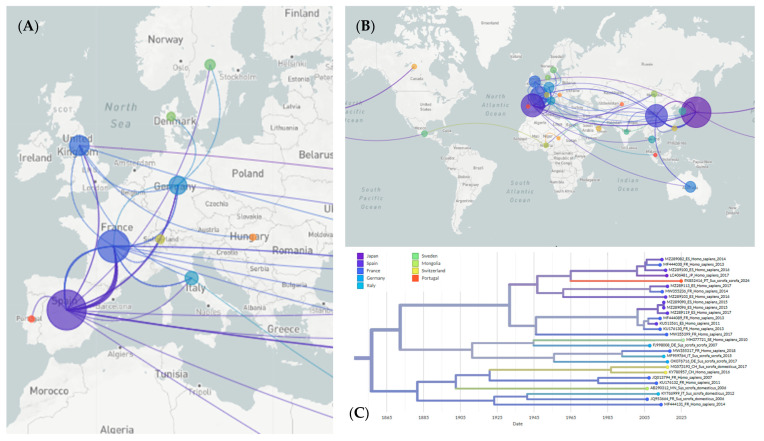
Auspice view of the HEV tree generated using the INSaFLU/Nextstrain pipeline and corresponding world map of the estimated transmission pathways. (**A**) Map showing transmission lines portraying potential viral dispersion routes in Europe; (**B**) Map showing transmission lines portraying potential global viral dispersion routes; (**C**) Expanded view of the tree branch used for phylogeographic analysis containing sequence PX852416. Colors represent country of origin and branch lengths represent time.

A phylogeographic analysis, conducted using the INSaFLU/Nextstrain pipeline, estimated that HEV-3m strain circulating in the wild boar population in Portugal likely originated in Spain. Frequent transmission events were observed between Spain and France, involving both HEV-3m subtype and HEV-3f subtypes. Several global transmission routes involving multiple transmission events were clearly identified, including those from China to Japan and France, from Japan to China, South Korea and Canada, from France to Spain and Germany, and from Spain to France, the United Kingdom, Germany and China. Other transmission routes were also identified, although these appeared sporadically rather than as consistent, systematic pathways (Figure 5). China, Japan, Spain and France were therefore identified as the most relevant HEV hotspots. It was estimated that HEV reached Europe in 1755 (CI: 1735–1770) from China, first being introduced into France. The HEV-3m strain circulating in Portugal was estimated to have arrived around 1967 (CI: 1962–1970), and it was likely introduced from Spain.

The inferred geographic origins of each HEV genotype were determined by examining the time-resolved phylogeographic reconstructions generated by the INSaFLU/Nextstrain pipeline. For each genotype-specific clade, the earliest ancestral node with an inferred geographical location was identified in Auspice; the corresponding country was then considered the most likely origin of that genotype. Based on this approach it was estimated that France was the origin of HEV genotypes 3m, 3ra, 3e, 3g, 3i, 3l, 3c and 3h, and that Spain was the origin of genotype 3f. Genotypes 3a, 3j, 3k, 3b and 6 likely originated in Japan, while genotypes 1, 4, 5 and 8 likely originated in China. Genotype 2 likely originated in India, and genotype 7 likely originated in the United Arab Emirates.

Root-to-tip regression performed in TempEst yielded a positive slope of 1.86 × 10^−3^ substitutions/site/year, but with a very low correlation (r = 9.4 × 10^−3^, R^2^ = 8.8 × 10^−5^) (Supplementary Figure S1). While Nextstrain phylogeographic reconstructions estimated the year 1671 (CI: 1642–1689) as the time to the most recent common ancestor (tMRCA), TempEst estimated the year 1864. These results suggest that this dataset lacks the temporal information necessary for reliable molecular clock calibration or precise dating of divergence events.

### 3.4. Recombination Analysis

A total of 42 recombination events were identified among the 263 sequences analyzed, ten of which were supported by at least three independent detection methods. No evidence of recombination was detected in the sequence obtained in this study (PX852416). Regarding other HEV-3m strains, one recombination event was detected by three different methods (RDP, GENECONV and Bootscan); however, it was not considered to be genuine as the two recombination points detected were both less than 100 nucleotides away from the ends of the genome. Another recombination event within HEV-3m sequences was detected in sequence MZ289113 (Spain); however, it was only detected by a single detection method (GENECONV), so it was not considered significant. The remaining recombination events, which occurred in sequences from genotypes HEV-3f (three sequences), HEV-3e (two sequences), and HEV-3ra, HEV-3h, HEV-3a and HEV-8 (one sequence each), were detected by three or more methods.

## 4. Discussion and Conclusions

The HEV positivity rate detected in Portuguese wild boar in this study was low (3.3%), aligning with the prevalence results of recent Portuguese studies, which reported values ranging from 0.8% to 2.8% [11,44,45]. Overall, the prevalence of HEV in Portugal remains lower than that described in several other European countries, including Hungary (12.2% in 2005–2006) [46], Sweden (15.1% in 2012–2015) [47], the Netherlands (7.5% in 2005–2008) [48] and Germany (18.2% in 2012–2013) [49]). By contrast, the prevalence of HEV in Spanish wild boar populations varies considerably by region, ranging from 2.2% to 60.0%, depending on both the location and the sampling period [17,18,50]. Spain, therefore, appears to be a major reservoir of HEV, with the highest prevalence levels consistently reported in south and southwest of the country. For example, 46.7% of the animals were infected in the Doñana National Park [17] and 22.3% in the Andalusia overall, with the infection rates peaking at 60.0% at the beginning of the hunting season in October and November [18]. These results contrast with the much lower prevalence in other Spanish regions, such as Barcelona (2.2%). The clustering of regions with a high prevalence of HEV in southwestern Spain, which borders Évora in Portugal, where positive cases have been detected, supports the hypothesis that HEV may be introduced to Portugal through the movement of wild boars across the border. These findings highlight the importance of continued surveillance of wild boar populations, particularly in regions bordering Spain, to track viral circulation and detect emerging strains.

However, the prevalence of HEV observed in wild boars should be interpreted with caution, as it can be strongly influenced by temporal and spatial factors. Seasonal fluctuations in virus circulation, local population density, age structure and habitat characteristics can all significantly impact detection rates. It is also known that wild boars from rural areas exhibit a significantly higher prevalence than those from urban environments [49]. Taken together, these patterns suggest that HEV prevalence estimates can be substantially impacted by the number of animals tested, the timing of sample collection, the characteristics of hunting areas (rural versus urban), and the degree of overlap between the wildlife and livestock habitats. These factors must therefore be carefully considered to avoid biased interpretations. The HEV strain fully sequenced in this study clustered within the HEV-3m genotype, a lineage composed predominantly of human-derived sequences. The conclusions regarding HEV-3m circulation in wild boars should be interpreted cautiously given that only one full genome was obtained. Despite this limitation, the detection of this genotype in wild boars in Portugal supports their role as potential reservoirs of zoonotic strains. Notably, sub-genotype 3m has also been identified in domestic pigs, suggesting that it can circulate across different host species. This finding supports the existence of interconnected transmission cycles between wild and domestic swine populations, which facilitate viral maintenance and dissemination. Such ecological overlap likely increases the likelihood of cross-species transmission and underscores the importance of domestic pigs as an additional reservoir. Taken together, these observations suggest the presence of a shared viral pool between wildlife and livestock, which has significant implications for zoonotic for the transmission of diseases to humans.

The results of the second phylogenetic tree, which was based on the 306-nt ORF1 fragment, further highlight the considerable genetic diversity of HEV circulating in Portugal. As already referred, given the limited phylogenetic resolution provided by the short sequence fragments, any inferences regarding subtype circulation at the national level should be made cautiously. The simultaneous detection of sub-genotypes 3e, 3f, and 3m in multiple regions of Portugal, without clear spatial or temporal clustering, suggests either repeated independent introductions or sustained endemic circulation. Identification of sub-genotype 3m in regions beyond the Alentejo region, including in the Norte and Centro regions, in both wild boar and domestic pigs, suggests that this subtype may be more widely distributed than the full-genome analysis alone would indicate. Moreover, the co-circulation of multiple sub-genotypes within the same regions over relatively short timeframes suggests a complex epidemiological landscape, which is probably the result of animal movements, interactions between wild and domestic hosts, and repeated viral introductions. However, the short length of the fragment on which the analysis was based limits phylogenetic resolution and the inference of detailed evolutionary relationships. Therefore, these findings should be interpreted with caution and, where possible, be supplemented with full-genome data.

The phylogeographic analysis revealed multiple transmission routes between European countries and similarly, among Asian countries, as well as sporadic transmission to other continents. This helps to explain how HEV strains evolve primarily within the same continent. While it provides a preliminary temporal context, the estimated dates are highly uncertain due to the extremely weak temporal signal identified by the TempEst analysis. Nevertheless, the INSaFLU/Nextstrain phylogenetic reconstruction provides useful geographical information and supports the theory that Spain is the most likely source of HEV introduction into Portugal.

Regarding the recombination analysis, the large number (42) of sequences being estimated to be of recombinant origin, despite only ten of these being supported by three or more independent methods, suggests that, although recombination has contributed to the genetic diversification of some HEV lineages, it has not been the primary driver of HEV diversification within the analyzed dataset. Furthermore, it appears to play an insignificant role in the evolution of the HEV-3m strains within this dataset.

It is important to note that phylogeographic and evolutionary inferences depend heavily on the availability and representativeness of sequence data, which vary considerably between countries. Nevertheless, the present phylogeographic analysis identifies two main HEV hotspots in Europe (France and Spain), as well as two others in Asia (Japan and China). The virus appears to have disseminated from these regions to most other geographic areas, including Portugal, which it most likely via Spain.

Despite the complete absence of other sequences from wild boars within the HEV-3m genotype, the estimated transmission route from Spain to Portugal, together with the several studies that used partial genome sequencing to identify HEV genotypes 3c, 3i, 3h, 3f, and 3m in wild boars from Spain [50,51,52], as well as and genotypes 3c, 3e and 3m in wild boars from Portugal [45,53], seems to suggest that the cross-border movement of wild boars from Spain to Portugal introduced this genotype to Portugal. However, these studies relied solely on short genomic fragments, typically within the ORF2 region. This approach completely ignores the role of recombination events in HEV evolution and consequently limits phylogenetic analysis. These methodological and sampling limitations hinder the comprehensive characterization of HEV diversity and transmission dynamics across the Iberian Peninsula.

These results emphasize the importance of a One Health approach by highlighting the zoonotic potential of the HEV strains circulating in wild boars. Therefore, monitoring HEV circulation in wildlife is important for both animal and public health. Integrating wildlife surveillance with human and livestock monitoring is essential for a better understanding of the risks of HEV transmission and for informing preventive strategies to mitigate zoonotic outbreaks.

This study addresses important knowledge gaps and provides valuable insights into several areas by combining complementary approaches, including nationwide epidemiological surveys, whole genome sequencing, and phylogenetic, phylogeographic and recombination analyses. Together, these approaches provide a thorough understanding of the circulation and evolutionary dynamics of HEV, as well as its potential for cross-species transmission. This underlines the importance of continued surveillance of wild boar populations and reinforces the need for a One Health approach.

## Figures and Tables

**Table 1:** Primers and probe sequences used for HEV detection by RT-qPCR and primers used for HEV whole-genome amplification by RT-PCR.

Reaction (s)	Primer Name	Sequence	Target Gene	Amplicon Size
HEV RT-qPCR	HEV-AB-F	5′-CGGTGGTTTCTGGGGTGA-3′	*Capsid/ORF3*	75 bp
	HEV-AB-R	5′-GCRAAGGGRTTGGTTGG-3′		
	HEV probe	5′-[FAM]TGATTCTCAGCCCTTCGC-3′		
HEV RT-PCR 1	Primer_HEV_Fw1	5′-CTCCTGGCATTACTACTGCCA-3′	*ORF1*	1188 bp
	Primer_HEV_Rv1	5′-TTYTGGGCRTGCTCAACCTC-3′		
HEV RT-PCR 2	Primer_HEV_Fw2	5′-CGGCTYATGACYTAYCTCCG-3′	*ORF1*	1358 bp
	Primer_HEV_Rv2	5′-RAGRAGVCGGCGRGTGCG-3′		
HEV RT-PCR 3	Primer_HEV_Fw3	5′-GTCHACATCTGGYTTYTCTAG-3′	*ORF1*	1360 bp
	Primer_HEV_Rv3	5′-TRAAVGTGGCVCCCTGGGC-3′		
HEV RT-PCR 4	Primer_HEV_Fw4	5′-GYGAGCTYATACGYGGGGC-3′	*ORF1*	1335 bp
	Primer_HEV_Rv4	5′-TAGCARTGTGCTATGATCGCC-3′		
HEV RT-PCR 5	Primer_HEV_Fw5	5′-TCYCTYGGCCTTGAGTGTGT-3′	*ORF1/ORF2/ORF3*	1491 bp
	Primer_HEV_Rv5	5′-TCTCRACAGAGCGCCARCC-3′		
HEV RT-PCR 6	Primer_HEV_Fw6	5′-TGGTGCCRAATGCYGTYGG-3′	*ORF3*	1019 bp
	Primer_HEV_Rv6	5′-ARAATGTYTTRGARTACTGCTG-3′		

## Data Availability

The data supporting the results of this study can be obtained by contacting the corresponding author; however, the right to privacy of the property owners will be respected.

## Supplementary Materials

The following supporting information can be downloaded at: https://www.mdpi.com/article/10.3390/pathogens15040430/s1, Supplementary Figure S1. Root-to-tip regression analysis of HEV sequences performed with TempEst. Gray dots represent PCV3 sequences, plotted according to its genetic divergence from the inferred root of the tree (y-axis, substitutions per site) against its sampling date (x-axis, in years). The black line represents the best-fit regression of root-to-tip distances over time. The slope of this line is 1.864 × 10^−3^ substitutions/site/year. The correlation coefficient is 9.356 × 10^−3^ and the coefficient of determination R^2^ equals 8753 × 10^−5^.

## Abbreviations

The following abbreviations are used in this manuscript:

bp	Base pairs
BIC	Bayesian Information Criterion
BSL3	Biosafety level 3
cDNA	Complementary DNA
CI	Confidence interval
DNA	Desoxyribonucleic acid
dNTPs	Deoxynucleotide triphosphates
DTT	Dithiothreitol
HEV	Hepatitis E virus
ML	Maximum likelihood
nt	Nucleotides
ORF	Open reading frame
PCR	Polymerase chain reaction
PBS	Phosphate-buffered saline
RNA	Ribonucleic acid
RT-qPCR	Reverse transcription quantitative polymerase chain reaction
SH-like aLRT	Shimodaira–Hasegawa-like approximate likelihood ratio test
tMRCA	Time to the most recent common ancestor
µL	Microliter
µM	Micromolar
*w*/*v*	Weight/volume
