# Supplementary material for: Wild Boars as a Reservoir of Zoonotic Hepatitis E Virus in Portugal with Full-Genome Evidence of Genotype 3m
Source: Pathogens. 2026 Apr 16;15(4):430. doi: 10.3390/pathogens15040430 (PMC13119151; doi:10.3390/pathogens15040430)
Supplement: Supplementary file 1 [file pathogens-15-00430-s001.zip › pathogens-4256175-supplementary.pdf]

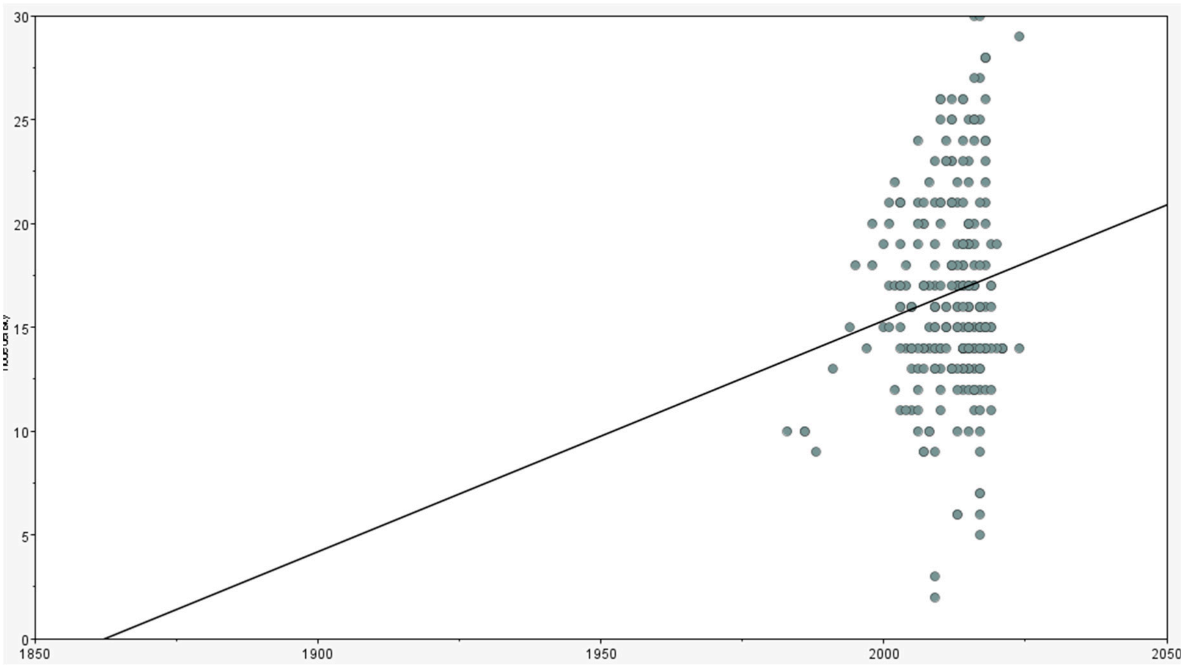

Supplementary Figure S1. Root-to-tip regression analysis of HEV sequences performed with TempEst. Gray dots represent PCV3 sequences, plotted according to its genetic divergence from the inferred root of the tree (y-axis, substitutions per site) against its sampling date (x-axis, in years). The black line represents the best-fit regression of root-to-tip distances over time. The slope of this line is 1.864×10^{-3} substitutions/site/year. The correlation coefficient is 9.356×10^{-3} and the coefficient of determination R^2 equals $8{,}753 \times 10^{-5}$.
